# Using the Five Core Components of Competency-Based Medical Education to Support Implementation of CBVE

**DOI:** 10.3389/fvets.2021.689356

**Published:** 2021-07-20

**Authors:** Kristin P. Chaney, Jennifer L. Hodgson

**Affiliations:** ^1^Department of Veterinary Integrative Biosciences, Texas A&M University College of Veterinary Medicine and Biomedical Sciences, College Station, TX, United States; ^2^Department of Population Health Sciences, Virginia-Maryland College of Veterinary Medicine, Blacksburg, VA, United States

**Keywords:** veterinary education, implementation, competency-based education, CBVE, assessment

## Abstract

In recent years, veterinary education has begun the transition to competency-based models, recognizing that, like medical education, our goals include improved patient and client outcomes and the importance of learner-centered methods in education. Given that implementation of competency-based veterinary education (CBVE) is still in its relative infancy across many veterinary programs, we stand to gain from a unified approach to its implementation. As a guideline, the five core components of competency-based medical education (CBME) should serve to ensure and maintain fidelity of the original design of outcomes-based education during implementation of CBVE. Identified the essential and indispensable elements of CBME which include 1) clearly articulated outcome competencies required for practice, 2) sequenced progression of competencies and their developmental markers, 3) tailored learning experiences that facilitate the acquisition of competencies, 4) competency-focused instruction that promotes the acquisition of competencies, and 5) programmatic assessment. This review advocates the adoption of the principles contained in the five core components of CBME, outlines the approach to implementation of CBVE based upon the five core components, and addresses the key differences between veterinary and medical education which may serve as challenges to ensuring fidelity of CBVE during implementation.

## Introduction

A new educational paradigm known as competency-based medical education (CBME) has emerged over the past 20 years and has been adopted by many healthcare training programs around the world. The goal of CBME is to develop “a health professional who can practice medicine at a defined level of proficiency, in accord with local conditions, to meet local needs” (1). This approach extends beyond teaching traditional medical knowledge and skills to include comprehensive training that prepares graduates for the complex roles of today's healthcare professionals (2).

As more and more health care programs shifted to adopt this model of education, it became clear there was often an inconsistent process for implementation of CBME among training programs due to either a lack of understanding of what constitutes a true competency-based program or an incomplete process of implementation. An unintended consequence of the inconsistencies in adoption left some programs believing CBME failed to meet expectations (3–5). In response to this concern, Van Melle et al. (3) used a two-step method to identify the essential and indispensable elements of CBME. This study identified five essential components that must be included in the implementation process to maintain fidelity of CBME. Fidelity, defined as the degree of exactness with which something is copied or reproduced, is considered critical for the success of implementing CBME. However, although this terminology infers rigidity or strictness in the rules of this model of education, it is important to recognize that CBME is a collection of pedagogical principles and approaches that are constantly evolving to meet the primary aim of achieving better outcomes for patients and learners (6). Furthermore, it is recognized there is a need for the experience, wisdom and expertise of local healthcare faculty to guide meaningful standardization where appropriate, while incorporating the flexibility and adaptability to meet the local needs of educational programs (7).

In recent years, veterinary education has begun the transition to competency-based models, recognizing that, like medical education, our goals include improved patient and client outcomes and the importance of learner-centered methods in education (2). Given that implementation of competency-based veterinary education (CBVE) is still in its relative infancy across many veterinary programs, we stand to gain from a unified approach to its implementation. As a guideline, the five core components of CBME should serve to ensure and maintain fidelity of the original design of outcomes-based education during implementation of CBVE. This review advocates adoption of the principles contained in the five core components of CBME, outlines approaches veterinary education could take to aid the implementation of CBVE based upon these core components, and addresses the key differences between veterinary and medical education that may serve as challenges to ensuring fidelity of CBVE during implementation.

## The Five Core Components

### Component #1: Clearly Articulated Outcome Competencies Required for Practice

As with any innovation, standardization of language is critical for adaptive change (8, 9). Developing a shared language with clear definitions that articulate key components is necessary for establishment of shared mental models across training programs and can be used to guide educators and learners toward teaching, learning, and assessing competencies. In CBME, articulation of outcomes occurs through development of a competency framework, defined as an organized and structured representation of a set of interrelated and purposeful competencies derived by the societal needs of clients and standards of care for patients (9).

### Component #2: Sequenced Progress

Sequencing learner progression within an educational program involves designing curricula and assessments that are more deliberate and explicitly developmental (10, 11). In medical education, the sequencing of learner progress has been supported through the use of milestones as a potential approach to help guide longitudinal, developmental assessment of the competencies, and to create educational outcome measures for individual learners (11, 12). Milestones are designated as defined, observable markers of an individual's ability along a developmental continuum (9). They are organized as narrative descriptions of abilities at various stages of professional development based on the Dreyfus model of skill acquisition (12, 13). Milestones may be used as a mechanism for formative assessment focused on learner improvement or summative assessment to help facilitate and support the ongoing development of individual learners and the continual quality improvement of training programs (11).

### Component #3: Tailored Learning Experiences

Tailoring learning experiences to the individual learner is a mechanism that allows time to be utilized as a resource by the learner. To achieve this goal, programs must consider removing time-based training and providing opportunities for learners to progress toward competency at their individual pace within a given timeframe. In medical education, an excellent example of time-variable training may be found in the Education in Pediatrics Across the Continuum (EPAC) program which was designed to advance learners from undergraduate medical education to graduate training based on competence rather than time spent in training (14). Originally developed as a pilot study and now operating as the standard of training for over 10 years, the central design features of this program include predetermined expectations of performance and transition criteria to ensure readiness for progression, including use of entrustable professional activities (EPAs).

### Component #4: Competency-Focused Instruction

There are inherent challenges associated with adoption of a new model of education, especially when faculty are comfortable in the perceived “stability” of the current system (15). As the movement toward outcomes-based education is initiated within a program, it is important ensure all faculty understand the curricular changes are based on evidence-informed approaches utilizing lessons learned from multiple research disciplines and fields (3, 7, 16).

To further mitigate skepticism and apprehension, it is essential that programs provide teachers with the faculty development necessary to prepare them to confidently meet the requirements of the new educational paradigm.

### Component #5: Programmatic Assessment

Programmatic assessment (PA) is an approach in which assessment is seen as a system, or a program, of assessments (17). Many educators consider PA as a “toolbox” in which training programs have a variety of assessment tools at their disposal to establish a more holistic evaluation of student performance. Each individual assessment in PA is viewed as a single data point for an individual learner that when aggregated, optimizes learning and guides progression. In addition to the numerous direct observations required to support progression and development of expertise, learners also require focused, specific feedback on their performance. PA is associated with the philosophical switch from the assessment *of* learning to the assessment *for* learning (18). To assist in the assessment for learning, CBME focuses on developing partnerships between the learner and the assessor based on the sharing of formative, coaching feedback that allows the learner to gauge their progress toward competence (19).

## Discussion

Five core components have been identified as necessary to ensure fidelity of implementation in CBME. We believe veterinary education can and should follow these five core components as more and more educational programs move to accept and transition to outcomes-based training. Adherence to these five core components can support veterinary programs during implementation and with modifications to address the differences and/or challenges associated with veterinary vs. medical education as well as local context and needs, we believe veterinary programs can successfully adopt and implement CBVE.

### Challenges/Modifications for Component #1

**Figure 1:**
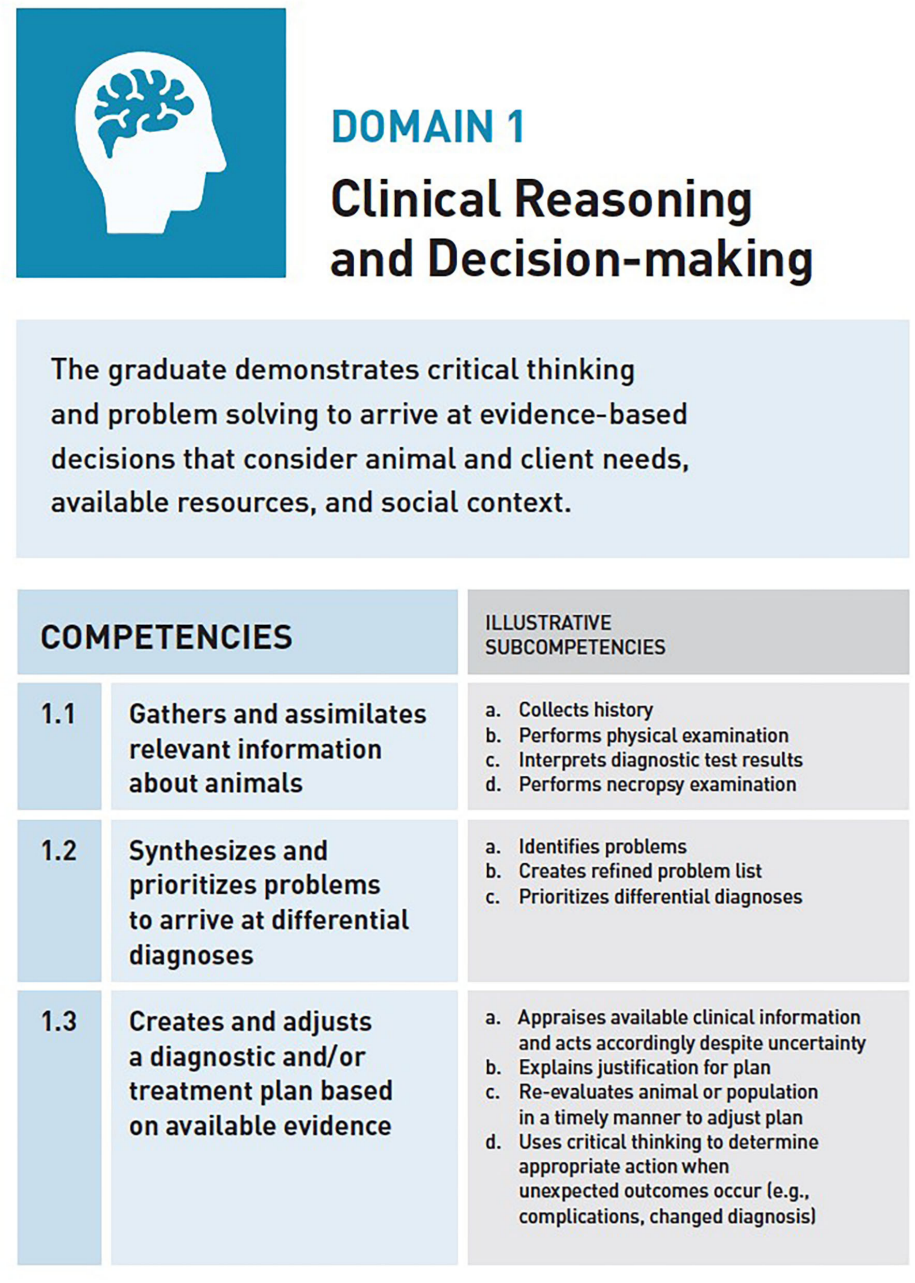
CBVE Domain of Competence 1: Clinical Reasoning and Decision-Making, an example/excerpt of the framework representing competencies and Illustrative subcompetencies.

A number of competency frameworks have been developed for veterinary education (2). The most recent is the CBVE Framework developed by the Association of American Veterinary Medical Colleges (AAVMC) (20). Designed as a guide for curricular development, course design, assessment planning, and program evaluation, the CBVE Framework is intended to be shared internationally across veterinary colleges in pursuit of global adoption of competency-based outcomes training in veterinary education. The framework identifies nine domains of competence and 32 competencies. Domains of competence are the highest level in CBME/CBVE frameworks and have been defined as broad distinguishable areas of competence that in the aggregate constitute a general descriptive framework for a profession (21). Domains of competence provide a general description of the spectrum of activities expected of a profession. Within each domain of competence, a set of associated competencies are identified and each represents an observable ability of a healthcare professional related to a specific activity that integrates knowledge, skills, values, and attitudes (22). Since competencies are observable, they can be measured and assessed to ensure their acquisition. Domains of competence and their associated competencies are considered the non-negotiable structures of the CBVE Framework. Their conservation across programs is what ensures maintenance of the framework as written and encourages the sharing between educational programs of information, assessments, and educational content related to the framework. The illustrative subcompetencies are provided as a representation of content that could be included within the broader competencies and are considered the component of the framework that may be customized by individual programs to support their local context and/or culture. An example of one of the CBVE domains, competencies and illustrative subcompetencies is provided in Figure 1.

The CBVE Framework is intended to be utilized widely without alteration to preserve the ability of programs to communicate and share assessments, content, and activities related to the outcomes as published. To better reflect local context and regional content, individual programs may choose to modify and adapt the framework by including additional illustrative sub-competencies or nested sub-competencies that are identified during the process of CBVE adoption and subsequent implementation (2, 23). Defining customized illustrative subcompetencies helps ensure curricular coverage of outcomes across the CBVE Framework and will be determined by the needs of the individual program. Educational programs can identify gaps in curricular content through collection of stakeholder feedback and curriculum mapping (24) to support development of illustrative subcompetencies and assist the curricular review and reform necessary to implement CBVE (25).

Recently, and following publication of the AAVMC CBVE Framework, multiple groups in veterinary education have developed, or are in the process of developing competencies related to specific subject area disciplines such as pharmacology, dentistry, informatics, emergency and critical care, and anesthesia. We offer a word of caution: while there are many 1,000s of competencies that could be developed in addition to those published with the CBVE Framework, this could cause the framework to become overwhelming for both educators and learners and act as a barrier for implementation. We advocate keeping the hierarchical structure of the CBVE Framework as published, leaving the newly developed “competencies” identified by different interest groups as illustrative subcompetencies to be mapped to individual CBVE competencies and associated domains of competence. The specific subcompetencies identified by each specialty group are intended for use within each group and not for the veterinary educational programs implementing the CBVE Framework. Again, maintaining fidelity of CBVE during implementation is critical for success of global adoption of outcomes-based education in veterinary medicine.

### Challenges/Modifications for Component #2

To assist sequenced training and learner progress in competency-based training in veterinary education, the AAVMC has published a series of milestones which align with the CBVE Framework (26) and are considered achievable by all students in CBVE training programs. These milestones can be used to guide curriculum development, help clinicians in developing workplace-based assessments such as in-training evaluation reports (ITERs), and to act as a roadmap for veterinary students along their learning continuum. Within CBVE, the milestones are sequentially designed with four anchors: novice, advanced beginner, competent, and proficient. The level of novice is expected to be achieved by the timepoint of entry into clinical training; the level of advanced beginner should be achieved during clinical training; and the level of competent to be reached by the time of graduation. The final milestone, proficient, is largely reserved for post-graduate training and achievement of veterinarians within the first year of practice. However, in some instances, this milestone may be achieved by learners during their educational training programs prior to graduation.

It should be noted that although the milestones as published are written with the novice level designed for entry into clinical service of the training program, moving the continuum of learning backwards into the preclinical program through the establishment of pre-clinical milestones can guide course development and establish outcomes related to discipline-specific or basic science course content. In this way, pre-clinical milestones can provide the outcomes for each competency at varying training stages of preclinical training essentially creating a roadmap for faculty as they design specific course and session learning outcomes to ensure learner achievement and acquisition of competencies prior to entry into clinics (i.e., Novice level).

**Figure 2:**
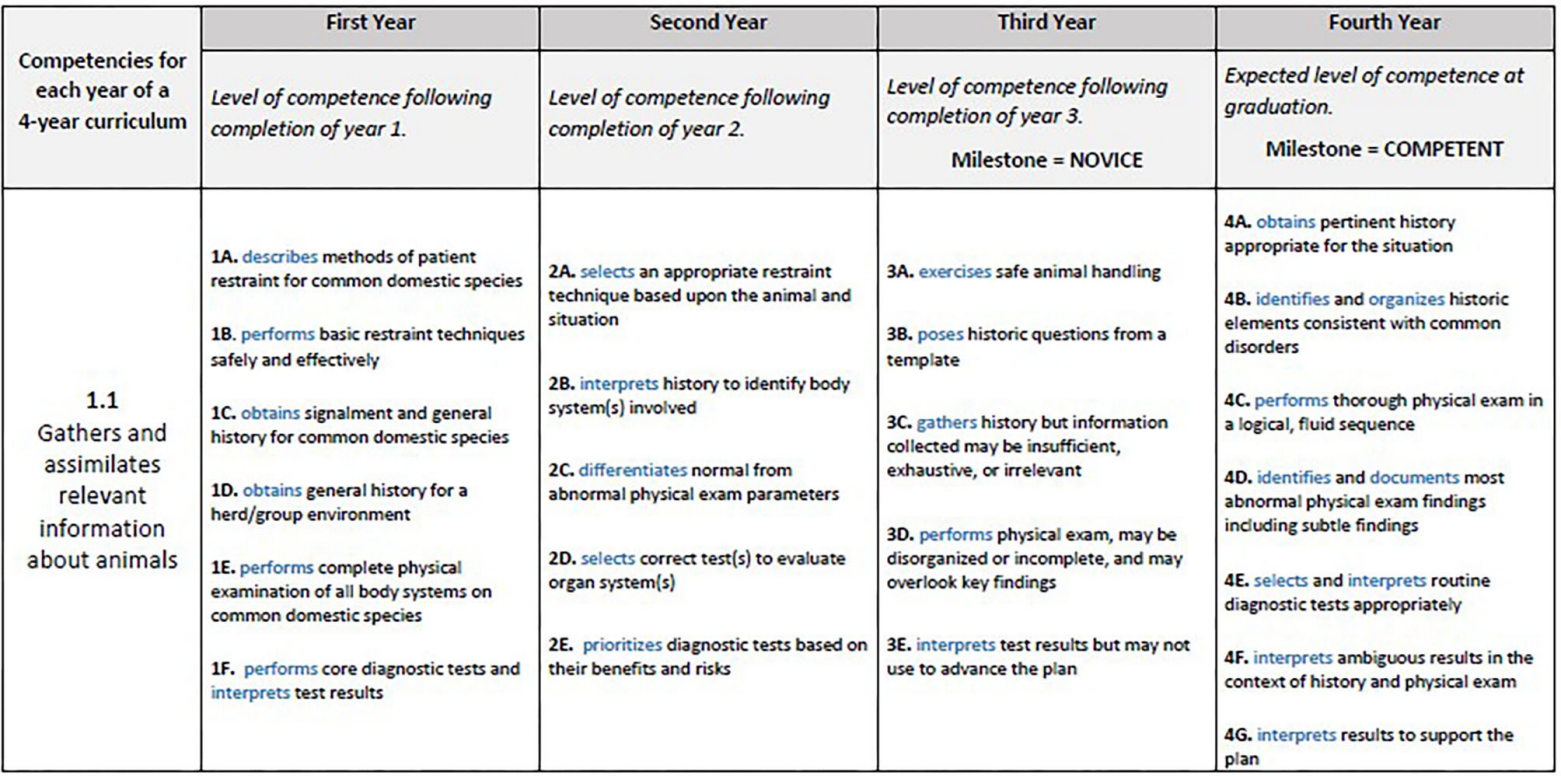
Clinical and preclinical milestones for CBVE Competency 1.1, Gathers and assimilates relevant information about animals.

A preclinical milestones example for CBVE competency 1.1 is provided in Figure 2, where these additional milestones have been written for outcome expectations in preclinical years; often occurring in years 1 and 2 in North American programs. To more thoroughly understand this example, follow the pathway of milestones that demonstrate a clinical student at the time of graduation (Competent milestone level 4.E), “selects and interprets routine diagnostic tests appropriately.” While this outcome is achievable by a clinical/senior veterinary student, it is critical to consider the building blocks or outcomes necessary to reach this milestone. By working backwards and in order to “select and interpret a diagnostic test,” a student must first be able to interpret test results prior to being able to use the results to advance the treatment plan (Novice milestone level 3E), and before a student is able to interpret tests to design treatment diagnostic plans, the student must first be able to select a correct test to evaluate a particular organ system and begin prioritizing the selection of a test based upon the risks or benefits of that test (Pre-clinical Year 2 milestone level 2E). And finally, following the pathway back to the entry level of the program, a student must first learn how to “perform core or basic diagnostics tests and interpret results” for the Pre-clinical Year 1 milestone level 1F (e.g., perform and interpret blood glucose on a glucometer). The idea of the “golden thread” and working backwards in this example also demonstrates the expectation that a student knows where/how to perform venipuncture (i.e., knowledge of anatomy) and why interpretation of blood glucose is important (i.e., knowledge of physiology). Application of basic sciences is a foundational component of medicine and surgery, and while not explicitly defined within the CBVE Framework, its importance is critical to the development of competencies within the educational program. The creation of preclinical milestones helps faculty in all disciplines of veterinary medicine understand where specific content discipline aligns with the competency framework. Use of preclinical milestones promotes improved integration and acceptance of CBVE across clinical and foundational science faculty and contributes to the success of implementation.

### Challenges/Modifications for Component #3

Arguably, tailoring learning experiences and altering the length of the training program may be the most difficult barrier for implementation of competency-based training in veterinary education. The reality of converting time into a resource is challenging due to the current guidelines outlined in the American Veterinary Medical Association's (AVMA's) Policies and Procedures (27) which requires the DVM curriculum to extend over a period equivalent to a minimum of four academic years. Furthermore, the consideration for tailoring learning based upon individual achievement of competency and the potential for expediting completion of the training program leaves many colleges and schools facing the reality of potential revenue lost across the veterinary program when the required time-in-training is no longer the same for all students. Despite this component being a potential challenge, it is important to recognize that using time as a resource provides students the opportunity to improve efficiency in their progression and achievement of proficiency in veterinary education.

Is it possible to develop a hybrid model of “time and achievement” for veterinary education? For example, using modules of practice, learners could spend the time necessary to ensure achievement of outcomes/competencies and when combined with a variety of assessment modalities throughout the curriculum [e.g., objective structured clinical exams (OSCEs), direct observation of skills (DOPs), entrustment scales for workplace-based activities/EPAs, etc.], proficiency of individual learners could be assessed and confirmed prior to graduation, which is currently used as the endpoint. In addition, it could be envisioned that completion of the series of “basic” or “core” modules would then allow learners to move to “advanced modules” or “career-focused” modules based upon professional interests. This type of hybrid model of CBVE could be supported using block-chain technology (28) by issuing certificates, badges, or other credentials confirming learner achievement. Student portfolios could be the repository for documentation of all achievements as a requirement for graduation and potentially used as support for employment post-graduation. This is clearly an area where additional consideration and discussion should direct future conversations. While we understand tailoring learning experiences may occur in specific sessions within a curriculum, we envision educational programs transitioning to allow/encourage individual students to create their own time-in-training program as the future of veterinary education.

### Challenges/Modifications for Component #4

We believe that successful implementation of CBVE related to competency-focused instruction relies upon faculty development opportunities to help align goals of the competency-based training program with the ability of educators to meet the goals of the new outcomes-based model. Providing opportunities for faculty to learn, practice, and reflect on their progress and development as educators within the new curricular model is essential for its acceptance and successful implementation. Assisting faculty development in the transition to competency-focused training should be a priority when implementing CBVE. The success of outcomes-based training relies on the transition of faculty to understand the difference between simply delivering content and that of developing a partnership of learning with trainees. Students must transition away from knowledge acquisition to knowledge application. These changes mirror recommendations regarding adoption of a growth mindset model in health professions education where the benefits to learners include increased reception to feedback while the benefits to educators focus on supporting collaborative relationships in safe learning environments (29).

Competency-focused instruction requires educators to understand a basic level of curriculum development and course design. The backwards course design model can assist faculty in building outcomes-based educational opportunities (30) and can be useful as a faculty development tool to ensure that the identification of program, course, or session-level outcomes occurs prior to the development of learning activities. This backwards design process maintains the focus of content delivery on the outcomes and acts as a mechanism to streamline curricula.

Another faculty development module used in medical education is the Fundamental Teaching Activities (FTA) Framework for CBME (31). These training guidelines were developed in response to the observation that preparation for clinical tasks in medical school and post-graduate training does not necessarily equip health care professionals to be teachers (32). To support and better prepare medical professionals to become strong teachers, the FTA guidelines are organized into three categories for faculty development in teaching and focus on the day-to-day work of educators within CBME: Clinical Preceptor, The Teacher outside the Clinical setting, and the Educational Leader (31). Similar to the use of milestones in CBME, each category activity is described along a developmental trajectory, demonstrating the potential for enhancing the teacher's abilities as she/he develops increasing expertise. Many of the same principles outlined in the FTAs apply to veterinary education and could be instituted to provide faculty development to support the transition to outcomes-based instruction. Similarly, a number of resources specific for veterinary education are available on the CBVE website (www.cbve.org).

### Challenges/Modifications for Component #5

Implementing programmatic assessment (PA) is one of the challenges currently facing physician-educators in CBME. Medical education programs that have designed successful PA strategies have included the following elements: (1) the creation of a program of assessments that represent all domains of competence within the competency framework, (2) the use of multiple raters/observers using multiple methods over time in formative-type low-stakes assessments, (3) the development of assessments that follow the shared mental model for progression toward achievement/competency, (4) the creation of assessments for learning using coaching feedback, (5) the design of assessments that include direct observation of activities performed by the learner in either simulated or workplace-based environments, and (6) the summative assessments that include multiple decisions by educators, using an aggregate to form a holistic vision of a learners achievement (e.g., using multiple “pixels” to form the picture of proficiency) (33).

The concept of EPAs, introduced by Ten Cate, provides the means to evaluate performance of everyday activities of a practicing clinician and supports longitudinal data collection on learner performance. EPAs are defined as an essential task of a discipline (profession, specialty, or subspecialty) that an individual can be trusted to perform without direct supervision in a given health care context, once sufficient competence has been demonstrated (9). EPAs operationalize competencies in a workplace-based setting because several competencies from multiple domains must be integrated simultaneously to execute EPAs (34, 35). Essentially, EPAs provide a readily applicable organizational structure that may be used by clinicians for observation, feedback, and assessment of the learner through application of multiple different assessment tools that evaluate a student's clinical performance (36). In veterinary education, a series of eight EPAs have been published (37, 38). Across medical and veterinary education, EPAs provide an excellent structure for use in the application of multiple different assessment tools that evaluate a student's clinical performance (e.g., entrustability scales, miniCEX, DOPS, etc.).

In many instances, collecting longitudinal data on trainee performance data is possible, but translating that data into a useful format may be problematic. It is understood that learners should have access to their longitudinal data to better understand how their performance compares to their cohort and informs their future goal-directed performance. Educators and administrators can use longitudinal data as a mechanism to assess learner competency across the continuum of the program and use the data to inform decisions related to trainee progress within the program. Determining the best process for utilizing data collected can be the rate limiting step in fully embracing PA in health care education.

In recent years, CBME competency committees have gained more favor in guiding decision making on learner progress across the training program (39, 40). Introduction of these committees could be a useful tool for educators in CBVE (41) as well as a mechanism to operationalize the PA data collected within a program. Designing the competency committee (e.g., faculty membership, terms of participation, etc.) and goals of the committee (e.g., how will the information be used within an individual training program, timing of feedback to the learner, who has access to the information, etc.) must be defined for maximal benefit from the competency committee. In order for a competency committee to easily access data, there is the need for software programs with dashboard capability that are readily available, affordable, and easy to use. We would argue that within veterinary education we should dedicate greater focus on PA and look to competency committees as a mechanism to utilize and operationalize PA data to assist educators, administrators, and trainees within each program.

## Conclusions

The five core components of CBME can serve as a checklist for veterinary programs who are implementing CBVE to ensure fidelity to the competency-based model of education as it was originally designed. The components do not need to be implemented simultaneously as each training program should consider their individualized starting point for redesigning or updating their current program to adopt competency-based outcomes. Despite the starting point for implementation, we maintain that all five components are required to maximize the benefits and standardize the use and sharing of information across veterinary programs. With modifications to address the differences and/or challenges associated with veterinary vs. medical education within the five core components we believe veterinary programs can successfully adopt and implement this emerging model of education.

## Author Contributions

All authors listed have made a substantial, direct and intellectual contribution to the work, and approved it for publication.

## Conflict of Interest

The authors declare that the research was conducted in the absence of any commercial or financial relationships that could be construed as a potential conflict of interest.
